# Management of maxillary impacted canines: A prospective study of orthodontists' preferences

**DOI:** 10.1016/j.jsps.2021.03.010

**Published:** 2021-03-31

**Authors:** Hamad Alqahtani

**Affiliations:** Orthodontic Department, Faculty of Dentistry, King Abdulaziz University, Jeddah, Saudi Arabia

**Keywords:** Preferences, maxillary impacted canine, palatal, open technique, closed technique

## Abstract

**Background:**

Maxillary canines are considered the most commonly impacted teeth, after the third molars. Orthodontists have different preferences on how to approach maxillary impacted canines (MIC). The objective of this article was to investigate orthodontists’ approach to managing MIC.

**Material and methods:**

A cross-sectional study comprising a comprehensive survey with 22 questions was sent to practicing orthodontists. This study explored the preferred diagnostic measures, surgical techniques, materials, and mechanics utilized to manage MIC;104 responses were returned.

**Results:**

Palatal impaction was reported to be encountered more often than labial impaction by 60% of the respondents. In 62% of the respondents, an oral and maxillofacial surgeon was the specialist preferred to perform the surgical exposure. In 66%, the choice of required surgical techniques was reported as a joint decision between orthodontists and other specialists who perform the surgery. Cone-beam computed tomography (CBCT) was reported to be the diagnostic x-ray of choice. The gold button with a chain was the preferred bonded attachment in 86% of cases. Less than half of the respondents bonded the attachments themselves during surgical exposure. A clear plastic retainer was the preferred retainer in 61% of the respondents, and 43% of the respondents tended to use a closed exposure technique. Coe-pak^TM^ was the preferred surgical pack for orthodontists who prefer an open exposure technique. Piggyback (double wire) was the preferable mechanic to move a palatally impacted canine.

**Conclusion:**

Our findings indicate that there are variations among orthodontists on how to manage MICs in terms of diagnostic methods, surgical management, materials, and mechanics.

## Highlights

• Children should be examined by the age of eight or nine years to assess for maxillary canine displacement and possible impaction.
• Maxillary impacted canines are best approached and managed through interdisciplinary approach involving a pediatric dentist, orthodontist, oral surgeon, and periodontist.
• The pediatric dentist can be the first specialist for early diagnosis and management of maxillary impacted canines, which can be achieved by facilitating the maxillary canine’s eruption and guiding it to its proposed location in the dental arch.

## 1 Introduction

Maxillary canines are crucial for the smile and facial esthetics. This is attributed to their critical location over the canine eminences which provides support to the upper lip and alar base. Adequate alignment of maxillary canines in addition to proper size and shape play an important role in smile beauty, correct smile line, and appropriate proportion of the upper anterior teeth. Moreover, maxillary canines have a great functional impact since they provide disocclusion of posterior teeth during excursive movements and they provide support to the overall dentition (Cruz, 2019).

Tooth impaction manifests as a result of tooth failure to erupt beyond the completion of its normal development pattern (Hamada et al., 2019). Maxillary canines are considered the most commonly impacted teeth, after the third molars. This impaction is more common in females and occurs bilaterally in 8% of the cases. Two thirds of maxillary canine impactions are located palatally (Luyten et al., 2020). Labial impactions primarily occur due to arch length discrepancy, whereas the etiology of palatal impaction is unknown (Manne et al., 2012). Two theories have been proposed to explain palatal impaction: the genetic and guidance theories. The genetic theory indicates that palatal impaction results from genetic factors and can present with other dental anomalies, such as the infraocclusion of primary molars, enamel hypoplasia, small maxillary lateral incisors, and aplasia of the second premolars (Peck et al., 1994, Baccetti, 1998). The guidance theory states that the roots of the upper lateral incisors serve as a guide for the eruption of the upper canines, in which they slide along their roots during an eruption. Thus, any interference with a guided eruption might result in a palatal impaction. These interference include supernumerary teeth, congenitally missing lateral incisors, transposition of teeth, and odontomas (Richardson and Russell, 2000).

In the course of the eruption of permanent teeth, aberrations in their eruption direction can lead to pressure on the roots of adjacent erupted teeth. These aberrations are more frequent in maxillary canines causing pressure on the root of erupted maxillary lateral incisors. Rafflenbeul detected root resorption of adjacent teeth using cone beam computed tomography (CBCT) in more than two thirds of a sample of 60 untreated children and adolescents who were diagnosed with maxillary impacted canines (Rafflenbeul et al., 2019). Another study has evaluated resorption of incisors after ectopic eruption of maxillary canines using CBCT. They found root resorption in 38% of the lateral incisors and 9% of the central incisors (Pasternak-Junior et al., 2018). Potential risk factors associated with root resorption of the adjacent teeth include enlarged canine follicle, initial proximity between the adjacent roots and the impacted canine, size and morphology of the lateral incisor, impaction severity, and female gender (Rafflenbeul et al., 2019).

It is crucial to diagnose the impaction of maxillary canines early to minimize treatment complexity, cost, and time (Margot et al., 2020). Thus, it is advisable to examine the patients by the age of eight or nine years to assess the displacement of canines from their normal position. Radiographic and clinical evaluations (palpation and visual inspection) can be used to investigate the possibility of canine impaction (Shapira and Kuftinec, 1998). Conventional radiographs, including panoramic, periapical, and occlusal radiographs, can help in the diagnosis of impacted canines, but they lack accurate assessment of the root resorption of lateral incisors (Rohlin and Rundquist, 1984). CBCT is more accurate in detecting the position of impacted canines in three dimensions and provides more information about the root resorption of adjacent teeth (Alqerban et al., 2009). Palpation can be done labially and palatally, using the index fingers to evaluate the location of erupting canines and to check for the presence of palpable bulges (Shapira and Kuftinec, 1998). If the canine bulge is absent after the age of 10 years, this outcome indicates that the canine is probably displaced or ectopically erupted (Ericson and Kurol, 1986). According to Bishara, the following signs can indicate canine impaction: retained primary canines or delayed eruption of permanent canines beyond the age of 14_15 years, palpable palatal bulge, no labial bulge, and distal tipping or delayed eruption of the lateral incisors (Bishara, 1992).

A maxillary impacted canine (MIC) can be managed early through an interceptive approach. This includes extraction of the primary canine, rapid maxillary expansion, and headgear utilization in order to provide enough space for the MIC to erupt. Other approaches that might suit older children and adults include surgical exposure (which can be done with or without orthodontic traction), transplantation, and extraction of the MIC. The appropriate choice depends on treatment duration, complexity, esthetic results, functional outcomes, and complications (Grisar et al., 2020, Izadikhah et al., 2020). Orthodontic traction of ectopic canines can lead to root resorption and alveolar bone loss of the canines and adjacent teeth. Thus, correct diagnosis and the use of CBCT are critical to obtain correct treatment plan and to decide the appropriate path for orthodontic traction to minimize its complications (Silva et al., 2017).

Periodontal status of the MIC is influenced by multiple factors including periodontal biotype, initial canine location, surgical technique, pre-existing mucogingival condition and orthodontic traction. Therefore, it is necessary to perform a thorough periodontal and CBCT evaluation to evaluate keratinized tissues, periodontal biotype, attached gingiva, alveolar bone crest position, and labial bone width (El et al., 2020). Surgical exposure and orthodontic traction of the MIC might affect periodontal condition due to periodontal damage caused by forced orthodontic movement of the impacted canines. Ectopic impacted canines have shown increased electric pulp testing scores, deep pockets, increased gingival bleeding and plaque accumulation, and reduction in surrounding bone level at the end of orthodontic treatment compared to the other canine with normal physiologic eruption (Caprioglio et al., 2019).

Orthodontists have different preferences on how to approach MIC. No studies have investigated orthodontists’ preferences on how to manage and deal with MIC. The aim of this study was to evaluate the preferred diagnostic measures, surgical techniques, materials, and mechanics utilized to manage MIC, as determined by orthodontists.

## 2 Material and methods

This cross-sectional study was conducted at orthodontic department, dental school, King Abdulaziz university in Jeddah, Saudi Arabia. A comprehensive questionnaire was sent to the participants through their emails. The target population of our study was practicing orthodontists from around the globe. A total of 337 orthodontists were contacted initially by email. Non-respondents were reminded twice with follow-up emails. Their email addresses were collected from orthodontic journals, different orthodontic associations and websites of orthodontic postgraduate programs. Responses were collected anonymously; 22 multiple-choice questions were included in the questionnaire. One or more answers could be selected for some of the questions. A Google Form was used to create the questionnaire. This form included a cover letter which explained the rationale for the study, number of questions, and author’s contact information. Then, participants were allowed to proceed to the questions.

In order to conduct this study, we followed the guidelines of dental school’s research ethics committee at King Abdulaziz university. Responses were kept confidential and anonymous and were only used to conduct this research. Participating orthodontists were provided with full explanation of the study in the first part of the survey. They consented and agreed to participate by starting the survey and submitting at the end.

This survey was conducted according to the guidelines reported by Burns et al (Burns et al., 2008). Questions were created through in-depth literature reviews following the “sampling to redundancy” method. All potential concepts and ideas about MIC were identified during the literature reviews to create the most appropriate questions. Then, questions with similar themes were categorized into three domains. The socio-demographic status of the orthodontists was explored in the first domain of this survey; participants were asked to state their gender, how long they have been an orthodontist, the country where they obtained orthodontic training, the country where they currently practice, and the type of practice.

The second domain included general questions regarding MIC. This domain explored the size of the brackets slot used, type of maxillary canine impaction encountered most often, preferred specialist to perform the surgical exposure and bonding, who makes the decision regarding the type of required surgical technique, access to CBCT, choice of diagnostic x-ray, bonding system and attachments used for canine bonding, whether space is provided within the arch before or after the surgical exposure, and the retention system.

The third domain related only to palatally impacted canines (PIC). This domain described the most often used surgical exposure technique, type of surgical pack utilized with an open exposure, amount of bone removed around the PIC, and mechanics used for the traction of the PIC.

Data analysis was performed using version 21 of Statistical Package for the Social Sciences (SPSS; IBM, Chicago, IL, USA). The data were shown as percentages and frequency. A chi-square test was used to examine the significance of differences. The p level was set at 0.05.

## 3 Results

One hundred and four responses were obtained from the participating orthodontists. Forty-seven percent of our participants were female. More than half of the participants had practiced orthodontics for more than ten years. Two thirds of the clinicians obtained their orthodontic training in North America and Europe. University-based practices were the most common form of practice among the respondents (Table1).

**Table 1:** Frequency distribution table showing descriptive statistics for demographic & biographic data related to the sample.

Variable	Responses	Response - {Number (%)}
Sample Size (n)		104
Gender	Female	49 (47.1)
	Male	55 (52.9)
Practice Years	1–5 Years	22 (21.2)
	6–10 Years	24 (23.1)
	11–15 Years	13 (12.5)
	More than 15 Years	45 (43.3)
Country from where education has been acquired	GCC	7 (6.7)
	North America	50 (48.1)
	Europe	28 (26.9)
	Others	19 (18.3)
Country where he/she practices	GCC	32 (30.8)
	North America	30 (28.8)
	Europe	25 (24.0)
	Others	17 (16.3)
Work Set up	Private	64 (61.5)
	Hospital	16 (15.4)
	University	72 (69.2)

GCC: The Gulf Cooperation Council for the Arab States of the Gulf; Bahrain, Kuwait, Oman, Qatar, Saudi Arabia, and the United Arab Emirates.

63% of the respondents preferred to use brackets with a slot size of 0.022 in.. Palatal impaction was reported more often than labial impaction by 60% of the respondents. Oral and maxillofacial surgeons were the preferred specialist to perform surgical exposure, as reported by 62% of the respondents. In 66% of the questionnaire, the choice of required surgical technique was reported to be a joint decision between orthodontists and specialist colleagues who perform the surgery. 91% of the clinicians had access to CT/CBCT, which was reported to be the diagnostic x-ray of choice. During surgical exposure, 86% of the participants reported that the gold button with a chain was the preferred bonded attachment. Less than half of the respondents bond the attachments themselves during surgical exposure. 90% of the participants tended to provide space within the arch for the impacted canine. A clear plastic retainer was the retainer of choice, as reported by 61% of the respondents (Table 2).

**Table 2:** Frequency distribution table showing descriptive statistics of general questions regarding MIC - Expressed as {Number (%)}

Variable	Category	Response-{Number (%)}
Which bracket SLOT size do you usually use?	0.018-inch	20 (19.2)
	0.022-inch	66 (63.5)
	Both	13 (12.5)
	Other	5 (4.8)
In your practice, what type of maxillary canine impaction do you encounter more often?	Buccal	7 (6.7)
	Palatal	63 (60.6)
	Both	34 (32.7)
For the impacted maxillary canines requiring surgical exposure, WHO do you prefer to do the exposure?	Yourself	1 (1.0)
	Periodontist	35 (34.6)
	Oral and maxillofacial surgeon	65 (62.5)
	Pedodontist	2 (1.9)
Who decides the required surgical technique?	Yourself	16 (15.4)
	The other specialist who received the referral	19 (18.3)
	As a joint decision between you and the other specialist	69 (66.3)
Do you have access to CT/CBCT?	No	9 (8.7)
	Yes	95 (91.3)
What do you USUALLY use as a diagnostic X-ray to perform a diagnosis and treatment plan for the impacted maxillary canines?	Intraoral Radiograph	40 (38.5)
	Panoramic Radiograph	76 (73.1)
	CBCT/CT	79 (76)
During surgical exposure, which ATTACHMENT do you prefer for canine bonding?	Threaded retentive pin	3 (2.9)
	Polycarbonate or gold crowns cemented onto the exposed crown	1 (1)
	Gold button with chain	90 (86.5)
	Lasso wires	3 (2.9)
	Stainless steel eyelet with twisted ligature wire	24 (23.1)
	Standard orthodontic bracket	18 (17.3)
	Titanium button with chain	11 (10.6)
During surgical exposure, who does the attachment BONDING?	Yourself	46 (44.2)
	The other specialist who performs the surgery	58 (55.8)
During surgical exposure, which BONDING SYSTEM do you prefer?	I do not do the bonding	42 (40.4)
	Three-steps system	27 (26)
	Two-step system	30 (28.8)
	One-step system	14 (13.5)
For impacted maxillary canines in general, do you use a different bracket system other than what you usually use for regular cases?	Yes	96 (92.3)
	No	8 (7.7)
When do you provide space within the arch for the impacted canine?	Before the surgical exposure	94 (90.4)
	After the surgical exposure	10 (9.6)
What type of retainers do you use for impacted canine cases?	Permanent lingual bonded retainer	51 (49)
	Hawley retainer	31 (29.8)
	Wraparound retainer	27 (26)
	Clear plastic retainer	64 (61.5)
	Other	4 (3.8)

Regarding PIC, 43% of the respondents tend to use a closed technique. For those who prefer an open technique, Coe-pak^TM^ was the preferred surgical pack. 51% of the clinicians preferred minimal bone removal during the exposure, just enough to bond the canine. In 51% of respondents, clinicians reported that they use the piggyback (double wire) as a preferred mechanic during the traction of PIC, followed by a ballista spring in 47% of the responses (Table 3).

**Table 3:** Frequency distribution table showing descriptive statistics of questions only relate to PIC - Expressed as {Number (%)}

Variable	Category	Response - {Number (%)}
Regarding PIC, which surgical exposure technique do you prefer?	Closed	45 (43.3)
	Open	23 (22.1)
	Both	36 (34.6)
Regarding open exposure, which surgical pack do you prefer?	I do not prefer open exposure	47 (45.2)
	Coe-pak^TM^	26 (25)
	Whitehead’s varnish	5 (4.8)
	Septoplast^TM^	1 (1.0)
	Glass-ionomer cement	7 (6.7)
	Other	18 (17.3)
During canine exposure, how do you prefer bone removal around the impacted canine	Bone to be removed completely around the crown until the Cemento-enamel junction	51 (49.0)
	Minimal bone removal enough to bond the canine	53 (51.0)
During traction of PIC, which mechanic do you use to move the canine?	Cantilever spring	25 (24)
	Ballista Spring	49 (47.1)
	Piggyback (double wire)	53 (51)
	Auxiliary arm from transpalatal arch	44 (42.3)
	Temporary anchorage devices (TADs)	34 (32.7)
	Buccal auxiliary spring	10 (9.6)
	K-9 spring	4 (3.8)
	Continuous super-elastic wire	29 (27.9)
	Active palatal arch	9 (8.7)
	Elastic Thread	9 (8.7)
	Other	5 (4.8)

Data were further analyzed to detect differences among variables in regard to gender, country of education, country of practice, and years of experience (Table 4, Table 5).

**Table 4:** Frequency distribution table showing Inferential statistics of general questions regarding MIC compared with baseline characteristics – Expressed – p value.

Variable		Gender	Practice Yrs.	Country of study	Country of Work
Which bracket SLOT size do you usually use?		0.616	0.623	0.135	0.098
In your practice, what type of maxillary canine impaction do you encounter more often?		0.076	0.858	0.052	0.089
For the impacted maxillary canines requiring surgical exposure, WHO do you prefer to do the exposure?		0.773	0.138	0.247	0.009^€^
Who decides the required surgical technique?		0.957	0.299	0.857	0.093
Do you have access to CT/CBCT? *		0.596	0.447	0.815	0.278
What do you USUALLY use as a diagnostic x-ray to perform a diagnosis and treatment plan for the impacted maxillary canines?	Intraoral Radiograph	0.013*	0.209	0.443	0.641
	Panoramic	0.597	0.091	0.017*	0.026*
	CBCT/CT	0.575	0.251	0.344	0.177
During surgical exposure, which ATTACHMENT do you prefer for canine bonding?	Threaded retentive pin	0.063	0.846	0.855	0.670
	Polycarbonate or gold crowns cemented onto the exposed crown	0.343	0.723	0.779	0.477
	Gold button with chain	0.732	0.193	0.000^¶^	0.000^¶^
	Lasso wires	0.627	0.256	0.162	0.093
	Stainless steel eyelet with twisted ligature wire	0.008^€^	0.651	0.054	0.034*
	Standard orthodontic bracket	0.787	0.744	0.302	0.074
	Titanium button with chain	0.907	0.089	0.034*	0.001^€^
During surgical exposure, who does the attachment BONDING?		0.188	0.243	0.000^¶^	0.000^¶^
During surgical exposure, which BONDING SYSTEM do you prefer?	I do not do the bonding	0.752	0.186	0.042*	0.000^¶^
	Three-steps system	0.567	0.692	0.338	0.032*
	Two-step system	0.953	0.064	0.467	0.010*
	One-step system	0.358	0.588	0.118	0.892
For impacted maxillary canines in general, do you use a different bracket system other than what you usually use for regular cases?		0.364	0.543	0.140	0.722
When do you provide space within the arch for the impacted canine?		0.848	0.272	0.001^€^	0.004^€^
What type of retainers do you use for impacted canine cases?	Permanent lingual bonded retainer	0.119	0.226	0.003^€^	0.000^¶^
	Hawley retainer	0.866	0.271	0.436	0.458
	Wraparound retainer	0.034*	0.059	0.398	0.050
	Clear plastic retainer	0.733	0.183	0.035*	0.418
	Other	0.366	0.368	0.942	0.187

*p value < 0.05; € p value < 0.01; ¶ p value < 0.001

**Table 5:** Frequency distribution table showing Inferential statistics of questions only relate to PIC compared with baseline characteristics – Expressed – p value.

Variable		Gender	Practice Yrs.	Country of study	Country of Work
Regarding PIC, which surgical exposure technique do you prefer?		0.432	0.469	0.357	0.226
Regarding open exposure, which surgical pack do you prefer?		0.030*	0.184	0.102	0.095
During canine exposure, how do you prefer bone removal around the impacted canine		0.000^¶^	0.032*	0.003^€^	0.004^€^
During traction of PIC, which mechanic do you use to move the canine?	Cantilever spring	0.720	0.262	0.023*	0.024*
	Ballista Spring	0.252	0.281	0.768	0.835
	Piggyback (double wire)	0.439	0.329	0.311	0.105
	Auxiliary arm from transpalatal arch	0.194	0.817	0.201	0.040*
	Temporary anchorage devices (TADs)	0.994	0.723	0.047*	0.001^€^
	Buccal auxiliary spring	0.127	0.956	0.295	0.094
	K-9 spring	0.255	0.694	0.027*	0.134
	Continuous super-elastic wire	0.558	0.691	0.582	0.744
	Active palatal arch	0.596	0.143	0.273	0.514
	Elastic Thread	0.024*	0.050	0.079	0.191
	Other	0.554	0.796	0.614	0.414

*p value < 0.05; € p value < 0.01; ¶ p value < 0.001

In regard to gender, female orthodontists showed statistically significant higher preference over males for intraoral radiograph as the choice of diagnostic x-ray, stainless steel eyelet as the choice of attachment for canine bonding, and higher preference for minimal bone removal during surgical exposure (0.013, 0.008, <0.001, respectively). On the contrary, males had higher preference over females for wraparound retainer as the choice of retainer, Coe-pak^TM^ as a surgical dressing, and elastic thread as a preferred method to move the impacted canine (0.034, 0.030, 0.024, respectively).

In respect to country of education, European-trained orthodontists had statistically significant higher preference for panoramic radiography, titanium button with chain, permanent lingual bonded retainer, and cantilever spring (0.017, 0.034, 0.003, 0.023, respectively). North American graduates showed higher preference for clear plastic retainer (0.035) and gold button with chain (<0.001). Orthodontist who got their training from the Gulf Cooperation Council for the Arab States of the Gulf (GCC) showed higher preference for gold button with chain (0.000), minimal bone removal during exposure (0.003), and space creation within the arch before the surgical exposure (0.001). Orthodontists who got their training from other countries had higher preference for bonding the attachment themselves during the exposure (<0.001), temporary anchorage devices (TADs) (0.047), and K-9 spring (0.027).

With regard to country of practice, orthodontists in North America showed statistically significant higher preference for oral and maxillofacial surgeon (0.009) and gold chain with button (<0.001). Orthodontists in Europe had a higher preference for panoramic radiography (0.026), stainless steel eyelet with twisted ligature wire (0.034), titanium button with chain (0.001), permanent lingual bonded retainer (<0.001), and cantilever spring (0.024). Orthodontists in GCC showed higher preference for gold button with chain (<0.001) and minimal bone removal (0.004). Clinicians in other countries had a higher preference for bonding the attachment themselves during the exposure (<0.001), bonding with three-step system (0.032), two-step system (0.010), minimal bone removal during surgical exposure (0.004), TADs (0.001), and space creation within the arch before the surgical exposure (0.004).

Finally, regarding years of experience, orthodontists who had practiced orthodontics for more than 15 years showed statistically significant higher preference for minimal bone removal during surgical exposure (0.032).

## 4 Discussion

Our study explored orthodontists’ preferences on how to deal with MIC regarding diagnostic methods, surgical approaches, materials, and mechanics. This survey comprised 104 responses; the data obtained represents the participants’ preferences. In regard to bracket slot size, a majority of the participants (63.5%) preferred 0.022 × 0.028 in. slot size compared to 0.018 × 0.025 in. (19.2%). This conforms with studies published in Brazil and the United Kingdom (Rampon et al., 2013, Banks et al., 2010). The majority of impactions reported by participants were palatal, which is in line with the literature (Ericson and Kurol, 1986, Alyami et al., 2020). Oral and maxillofacial surgeons were the preferred specialists to perform surgical exposure. The choice of surgical technique was generally described as a joint decision between the orthodontist and the specialist to perform the surgery. In contrast to our results, Naoumova et al. found that a pedodontist was the preferred specialist for performing the exposure, and the orthodontist selected the appropriate technique (Naoumova et al., 2018).

Radiographic examination plays a crucial role in the diagnosis and treatment of impacted canines. Alqerban et al. contrasted the impact of CBCT vs. panoramic x-ray for the surgical management of impacted canines (Alqerban et al., 2013). Alqerban et al. found no significant difference in pre-surgical treatment planning regarding treatment choice and surgical approach. Also, CBCT enhanced the orthodontist’s confidence level concerning canine location, presence of root resorption, and treatment planning. In our study, CBCT was the diagnostic x-ray of choice for most of our participants, followed by OPG. Moreover, most of our participants had access to CBCT.

The intended direction of the orthodontic traction of an impacted canine determines the preferred site for the attachment’s bond. For instance, if the impaction is located palatally and in line with the arch, the mid-buccal position of the attachment is favorable, and direct ligation to the archwire can move the tooth toward its final position. Moreover, it is important to distinguish the buccal and palatal aspects to prevent bonding to the wrong surface, which might lead to a full rotation of the canine when it reaches the archwire (Becker and Chaushu, 2015). If the canine is located mesially to the root of the upper lateral incisor, it must be pulled away in a vertical and/or posterior direction. Thus, it is preferred to place the attachment in the palatal aspect. When the canine is clear of obstruction, the canine can then be pulled directly toward the archwire. It is crucial to determine the precise location of the attachment and the direction of pull by the orthodontist to move the canine successfully. This procedure becomes highly reliable when both orthodontists and other specialists work as a team. A gold button with a chain was the preferable attachment for most participants. More than half of them reported that they do not bond the attachments themselves. This bonding was reported to be performed by other specialists who perform the surgical exposure.

Many surgical techniques have been advocated to expose MIC. These techniques are classified into open and closed procedures. Regarding labial impaction, Kokich proposed three surgical techniques: gingivectomy, apically positioned flap, and closed eruption techniques (Kokich, 2004). The choice depends on the labiolingual position, vertical position in relation to the mucogingival junction, mesiodistal position, and the amount of keratinized gingiva. Any technique can be used with a coronally-positioned impacted canine with adequate width of keratinized tissue (2 to 3 mm of attached gingiva). When the impacted canine is located apically to the mucogingival junction and mesiofacial to the root of the lateral incisor, an apically positioned flap is indicated. This technique is also indicated in the case of insufficient attached gingiva around the impacted canine. A closed technique is indicated if the canine is deeply impacted apically to the mucogingival line (Hamada et al., 2019, Kokich, 2004).

Regarding palatal impaction, open and closed techniques can be utilized to perform the exposure (Grenga et al., 2021). The open exposure is executed by removing the tissue over the impacted tooth and covering the area with a surgical pack for ten days. The tooth is allowed to erupt spontaneously. An attachment can be placed later to pull the exposed tooth to the arch (Parkin et al., 2017). The main advantage of the open technique is short surgical duration and eruption time. The major disadvantage is prolonged postsurgical recovery and sensitivity (Luyten et al., 2020).

The closed technique consists of reflecting a full-thickness flap of palatal mucosa, followed by follicle removal. An attachment is then bonded to the exposed crown, and a chain or a wire is placed. Then, the flap is sutured back to its original place. Traction can be applied shortly after the surgery to bring the canine to its planned position (Parkin et al., 2017, Kokich, 2004). The major advantages of the closed technique are fewer postsurgical complications, quicker recovery, and reduced postsurgical pain and discomfort. The main drawbacks include longer surgical duration, bond failure because of contamination, and this technique being more sensitive and complex (Luyten et al., 2020, Alberto, 2020). Regarding treatment outcomes, there is no evidence whether one technique is more efficient than the other (Parkin et al., 2017). 43% of our respondents preferred the closed technique exclusively, while 34% preferred both techniques. In contrast to our study, Naoumova et al. found that 48% of orthodontists who participated in the survey preferred both techniques, and 28% used the open technique exclusively (Naoumova et al., 2018). Moreover, Boyd reported an equal distribution of respondents favoring one technique only (Boyd, 1984).

Many surgical packs have been used in open exposure procedures, including glass-ionomer cement, Coe-pak^TM^, Whitehead’s varnish, and Septoplast^TM^. These packs are applied to the surgical site and removed after one to two weeks (Gharaibeh and Al-Nimri, 2008, Parkin et al., 2012, Cassina et al., 2018). Most of our participants who prefer open exposure tend to use Coe-pak^TM^ as a surgical dressing. In contrast to our study, Naoumova et al. reported that 72% of their participants tend to use glass-ionomer cement as a surgical pack (Naoumova et al., 2018). Glass-ionomer cement can bond to the tooth surface without etching and is reported to have fewer complications and less post-operative discomfort, even if it is placed for a long time (Nordenvall, 1992).

To uncover deeply impacted canines, an adequate amount of bone has to be removed to facilitate bonding and traction. Bishara recommended that only enough bone removal should be done to allow for bonding without an intentional exposure of cemento-enamel junction (Bishara, 1998). The relationship between the magnitude of bone removal and the resultant bone loss around the impacted tooth has been evaluated by McDonald and Yap. They found greater bone loss after orthodontic treatment in the case of greater initial bone removal (McDonald and Yap, 1986). In the same context, it was reported that cases managed with greater bone removal had 5.4% less bony support than cases managed with less bone removal (Kohavi et al., 1984). A major finding in our study was that more than half of the participating orthodontists preferred minimal bone removal of just enough to bond the impacted canine.

Post-treatment retention of the impacted canines should be planned carefully to minimize relapse and unwanted movement. Impacted canines have been investigated by Becker et al. regarding post-treatment alignment after finishing orthodontic treatment. Becker and colleagues found more spacings or rotations in 17.4% of the cases on the impacted side compared to 8.7% on the control side. Becker and colleagues concluded that the ideal alignment on the control side was as twice that on the impacted side (Becker et al., 1983). Woloshyn et al. reported significant post-treatment alignment problems, such as lingual displacement, intrusion, and rotation in 40% of impacted teeth compared to 91%, which had a normal appearance on the control side (Woloshyn et al., 1994). Therefore, a bonded fixed retainer or fiberotomy is indicated to prevent or minimize rotational relapse (Bishara, 1998). Interestingly, most of our participants preferred a clear plastic retainer, followed by a permanent lingual retainer.

Different methods have been invented to pull the impacted canine to its desired position. These methods include the K-9 spring, the ballista spring, the cantilever spring, active palatal arch, elastomeric chains or threads, and piggyback (double archwire) (Fischer et al., 2000, Kalra, 2000, Jacoby, 1979, Becker and Zilberman, 1978, Iancu Potrubacz et al., 2018). The piggyback wire was the most preferable traction method followed by the ballista spring.

Open and closed surgical techniques have been compared for patient-reported outcomes and perceptions such as pain experience and analgesic consumption. Gharaibeh and Al-Nimri found a similar postoperative pain after open and closed surgical procedures (Gharaibeh and Al-Nimri, 2008). Similarly, Parkin et al reported no statistically significant difference in pain duration or consumption of pain killers between both techniques (Parkin et al., 2012b). In contrast, Chaushu et al reported significantly higher postoperative pain and analgesic consumption among patients who had open surgical technique (Chaushu et al., 2005). A similar conclusion was reached by Björksved et al who found higher post-operative pain in the open surgical group. However, there was no statistically significant difference regarding analgesic consumption between both approaches (Bjorksved et al., 2018).

Many studies have explored the efficacy of different analgesics on reducing pain in orthodontic treatment mainly during insertion of orthodontic separators or archwire placement. Naproxen and ibuprofen provide stable analgesic effect that could peak at 6 h. Acetaminophen has an analgesic effect that rises steadily from 2 through 24 h. In comparison to acetaminophen and ibuprofen, Naproxen provides more potent analgesic effect which lasts to 24 h (Cheng et al., 2020). No studies have investigated the effect of different pain killers on the relief of pain associated with surgical exposure of MICs. Furthermore, future studies need to be conducted to analyze the orthodontists’ preferences in regard to presurgical and postsurgical analgesics and antibiotics in MIC cases that require surgical exposure.

This study has major strength points. In order to create this survey, we followed the guidelines for the design and conduct of self-administered surveys of clinicians published by Burns et al (Burns et al., 2008). We addressed our research questions with 22 items using “sampling to redundancy”. Our questions were unbiased and nonjudgmental and were easy to interpret and understand. Most of them contained <20 words. Despite these strengths, our study has limitations. Although our survey was sent online to many orthodontists around the globe, the response rate did not yield a large sample size. This might affect the generalizability of our results. This issue can be improved in future studies by including more variables, using shorter questions, and conducting this study over longer duration. Furthermore, the validity and reliability of the questionnaire have not been established.

## 5 Conclusions

Our findings indicate that there is no agreement among orthodontists on how to manage MICs concerning diagnostic methods, surgical management, materials, and mechanics. Guidelines based on scientific evidence are needed to guide practitioners for a common protocol to manage MIC.

## Declaration of Competing Interest

The author declares that he has no known competing financial interests or personal relationships that could have appeared to influence the work reported in this paper.
